# Efficacy and Tolerability of Telmisartan Plus Amlodipine in Asian Patients Not Adequately Controlled on Either Monotherapy or on Low-Dose Combination Therapy

**DOI:** 10.1155/2014/475480

**Published:** 2014-02-27

**Authors:** Dingliang Zhu, Pingjin Gao, Nobutaka Yagi, Helmut Schumacher

**Affiliations:** ^1^Ruijin Hospital, Shanghai Jiaotong University School of Medicine, Shanghai Institute of Hypertension, 197 Ruijin Road, Shanghai 200025, China; ^2^Nippon Boehringer Ingelheim Co., Ltd , 2-1-1, Osaki, Shinagawa-ku, Tokyo 141-6017, Japan; ^3^Boehringer Ingelheim Pharma GmbH & Co. KG, Binger Straße 173, 55216 Ingelheim, Germany

## Abstract

*Objective*. To evaluate the efficacy and safety of the telmisartan plus amlodipine (T/A) single-pill combination (SPC) in Asian patients with hypertension whose blood pressure (BP) was not adequately controlled on either monotherapy or on low-dose combination therapy. *Patients and Methods*. Data are presented from five Boehringer Ingelheim-sponsored phase 3, double-blind, 8-week, studies: two studies in nonresponders to amlodipine (data pooled for amlodipine), two studies on nonresponders to telmisartan (pooled data), and one on nonresponders to low-dose T/A SPC. *Results*. After 8 weeks' treatment, mean reductions from the reference baseline in diastolic BP (DBP; primary endpoint), systolic BP (SBP), and SBP, DBP goal, and response rates were higher with the T/A SPC than respective monotherapies. The T80/A5 SPC resulted in greater reductions in DBP and SBP, and higher DBP goal and response rate than the low-dose T40/A5 SPC. Peripheral edema incidence was low (amlodipine 0.5%, telmisartan 0.0%, and T/A SPC 0.7%). *Discussion and Conclusion*. In Asian patients whose BP is not adequately controlled with telmisartan or amlodipine monotherapy, T/A SPC treatment results in greater BP reduction, and higher DBP and SBP goal and response rates. The safety and tolerability of the T/A SPC are comparable to those of the respective monotherapies and consistent with those reported in previous studies.

## 1. Introduction 

In the Asia Pacific Cohort Studies Collaboration, up to 66% of some subtypes of cardiovascular (CV) disease in the Asia Pacific region were attributed to hypertension [1]. High blood pressure (BP) was associated with an increased risk for CV diseases, stroke, and heart disease among the Japanese [2–4] and Chinese population [5]. The Evidence for Cardiovascular Prevention from Observational Cohorts in Japan Research Group (EPOCH-JAPAN) study reported an approximate 20% hypertension prevalence in the Japanese population [6]. Similarly, in the Chinese population, overall hypertension prevalence has been reported to be 21.5% [7], with a higher prevalence (59.4%) reported in the elderly [8]. In Asians in general, the BP control rates are low, and there is a greater association between BP and CV risk [9].

Rapid and sustained BP goal achievement is important to reduce CV risk. At least 75% of patients with hypertension require combination therapy to achieve early BP goal [10], and guidelines recommend fixed-dose single-pill combinations (SPC) for their simplicity of treatment, convenience, and cost effectiveness [11, 12]. SPCs improve treatment adherence, resulting in better BP control and long-term CV risk reduction [13–15]. Treatment with SPCs has also resulted in significant annual cost savings [16, 17]. Significant improvement in compliance and nonsignificant beneficial trends in BP and adverse effects have been observed with SPCs compared with free drug combinations [18].

A renin-angiotensin system (RAS) inhibitor plus a calcium channel blocker (CCB) combination is recommended as a rational combination for hypertension treatment [11, 12, 19, 20] and is also the preferred combination in patients at high CV risk and those with evidence of renal disease, due to its CV and renoprotective benefits [21–23]. Telmisartan is the only angiotensin receptor blocker (ARBs) with demonstrated CV risk reduction similar to the angiotensin-converting enzyme (ACE) inhibitor ramipril, in patients at high CV risk [24] and provides superior and consistent BP reductions over 24 hours and beyond compared with other ARBs [25] and antihypertensive agents [26]. A substudy of ONTARGET (the Ongoing Telmisartan Alone and in Combination with Ramipril Global Endpoint Trial) and TRANSCEND (Telmisartan Randomized Assessment Study in ACE Intolerant Subjects with Cardiovascular Disease) that compared the tolerability of telmisartan and ramipril in Asian versus non-Asian patients showed that the advantage of better tolerability with telmisartan than ramipril was greater among Asian than non-Asian patients [27]. The telmisartan plus amlodipine (T/A) SPC (MICAMLO) is approved in Japan at the dose combinations of telmisartan 40 mg/amlodipine 5 mg (T40/A5) and telmisartan 80 mg/A5 (T80/A5) for the treatment of hypertension in patients not controlled on either monotherapy. The T/A SPC is approved for the treatment of hypertension as initial therapy, add-on therapy, or replacement therapy in Vietnam (doses: T40/A5 and T80/A5) and Malaysia (T40/A5, T40/amlodipine 10 mg [A10], T80/A5, and T80/A10) and as add-on or replacement therapy in Taiwan (doses: T40/A5, T40/A10, T80/A5, snf T80/A10) and Korea (doses: T40/A5 and T80/A10 as add-on or replacement therapy; T80/A5 and T40/A10 as add-on therapy).

The objective of this analysis was to evaluate the efficacy and safety of the T/A SPC in Asian patients with hypertension whose BP was not adequately controlled on either amlodipine or telmisartan monotherapy or on low-dose combination therapy.

## 2. Patients and Methods

### 2.1. Studies

Five Boehringer Ingelheim-sponsored studies assessed the T/A combination in Asian patients with hypertension: two studies on nonresponders to amlodipine monotherapy (A5 nonresponder study 1 [NCT00558064], A5 nonresponder study 2 [28] [NCT01103960]), two studies on nonresponders to telmisartan monotherapy (T40 nonresponders study [NCT00550953], telmisartan T80 nonresponders study [NCT01222520]), and one study on nonresponders to low-dose T/A combination (T40/A5 nonresponders [NCT01286558]) (Table 1).

### 2.2. Patients

In all studies, patients included were those with essential hypertension, at least 20 years old (≥18 years old in the A5 nonresponder study 2), and of either sex. In all studies, patients with secondary hypertension, any significant or unstable systemic disease, and previous experience of symptoms characteristic of angioedema during treatment with ACE inhibitors or ARBs and women who were pregnant, breastfeeding, or planning to become pregnant were excluded. Additional exclusion criteria at screening were treatment with four or more antihypertensive medications in the T40 nonresponder study and A5 nonresponder study 1; and three or more antihypertensive medications in the T80 nonresponder study and nonresponder to low-dose combination study. In all studies, those noncompliant with study medication during the open-label run-in period (defined as having taken <80% or >120% of prescribed medication, based on pill count) were also excluded. The BP inclusion and exclusion criteria are provided in Table 1.

All five studies were carried out in compliance with the protocol, the principles laid down in the Declaration of Helsinki, the International Conference on Harmonisation's Harmonised Tripartite Guidelines for Good Clinical Practice, and applicable regulatory requirements. The study protocols were reviewed by independent ethics committees or institutional review boards at each study site, and all patients provided written informed consent before entering the studies.

### 2.3. Study Design

All five studies were phase 3, prospective, multicenter, randomized, active control, double-dummy, double-blind, and parallel group studies. In all studies, patients who did not achieve diastolic blood pressure (DBP) goal <90 mm Hg (<80 mm Hg in the nonresponder to low-dose combination study) with treatment during the open-label run-in period were randomly allocated in a 1 : 1 ratio to double-blind treatment for 8 weeks. The study details are provided in Table 1.

### 2.4. Efficacy Assessments

In all five studies, BP and pulse rate were measured in the morning, approximately 24 hours after the last dose. Seated BP was measured at each visit using a standard validated and calibrated traditional, manual cuff sphygmomanometer, after the patient had rested in a seated position for approximately 5 minutes. Blood pressure was measured in the same arm, using the arm with the higher BP value as determined at the screening visit, and preferably by the same person at all study visits. The accuracy of BP measurements was increased by taking the mean of three consecutive measurements approximately 2 minutes apart. The seated pulse rate was measured during the 2-minute interval between the second and the third BP measurements.

### 2.5. Efficacy Endpoints

In all the studies, the primary endpoint was the reduction from the reference baseline in mean seated DBP at trough (24 hours postdosing) after 8 weeks of double-blind treatment. The reference baseline was defined as the BP value measured at the end of the open-label run-in period, immediately before first dosing in the double-blind treatment period. The secondary endpoints, which were the same for all studies, included change from reference baseline in seated trough systolic blood pressure (SBP); the proportion of patients achieving BP goal (mean seated trough BP < 140/90 mm Hg); DBP goal attainment (mean seated trough DBP < 90 mm Hg); SBP goal attainment (mean seated trough SBP < 140 mm Hg); the proportion of patients achieving DBP response (mean seated trough DBP < 90 mm Hg or DBP reduction ≥ 10 mm Hg); and the proportion of patients achieving SBP response (mean seated trough SBP < 140 mm Hg or SBP reduction ≥ 20 mm Hg [≥15 mm Hg in A5 nonresponder study 2]). Efficacy endpoints were assessed after 4 and 8 weeks' treatment or at last through observation during the double-blind treatment period.

### 2.6. Safety Assessments

In all five studies, physical examination, laboratory testing, and 12-lead electrocardiogram assessment were carried out at screening, at randomization, and at the end of the study or at early withdrawal. Pulse rate and adverse events (AEs) were recorded at all visits.

### 2.7. Statistical Analyses

In the confirmatory analysis of all five studies, DBP and SBP reduction after 8 weeks of double-blind treatment was analyzed using an analysis of covariance (ANCOVA) model with treatment as fixed effect, center as random effect, and reference baseline as covariate; in the A5 nonresponder study 2, country (instead of center) was included. Last trough observation carried forward was used to impute missing data for the endpoints involving seated trough BP measurements. Safety endpoints were descriptively summarized.

The statistics of this overview are purely descriptive. The full analysis set, which included all patients who took at least one dose of investigational treatment and for whom a reference baseline measurement and at least one BP measurement on randomized treatment were available, was used for demographic and baseline characteristics and efficacy analysis. The treated set, which included all randomized patients who took at least one dose of investigational treatment, was used for safety analysis. SAS version 9.3 (SAS Institute Inc., Cary, NC, USA) was used for the analyses.

Data from the five studies are presented in three groups—(1) nonresponders to A5: data from the two amlodipine nonresponder studies (data pooled only for the A5 group from the two studies); (2) nonresponders to T40 or T80: data from the two telmisartan nonresponder studies; and (3) nonresponders to the T40/A5 combination: data from the one study on T40/A5 nonresponders.

## 3. Results

### 3.1. Patients

A total of 1542 patients were enrolled across the five studies. The demographic and baseline characteristics of the patients were similar in the three study groups. The patients' mean age was in the range of 52–57 years; most (>60%) patients were <60 years of age, and most patients were men in each of the treatment subgroups included in the three study groups (Table 2). All patients were Asian, and >84% did not have a history of diabetes. The mean duration of hypertension was 6.6–8.6 years. Mean SBP/DBP at time of randomization (reference baseline) was >140/>95 mm Hg in the monotherapy nonresponder trials and >130/90 in nonresponders to the low-dose combination (Table 2).

### 3.2. Efficacy

**Figure 1:**
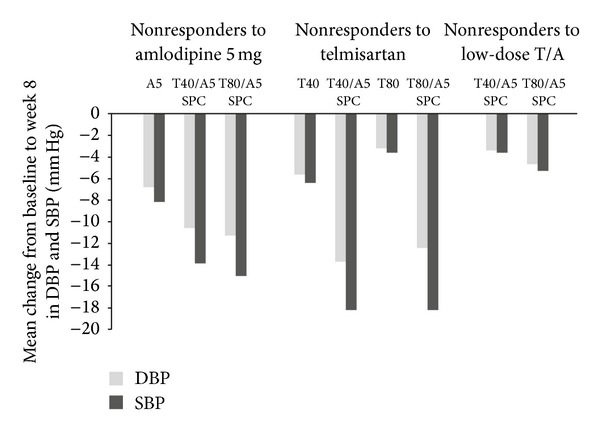
Change from baseline in DBP and SBP in the three groups after 8 weeks' treatment with T/A combination. Baseline indicates reference baseline BP value, which was measured immediately before first dosing in the double-blind treatment period. A5: amlodipine 5 mg; BP: blood pressure; DBP: diastolic blood pressure; SBP: systolic blood pressure; SPC: single-pill combination; T/A: telmisartan/amlodipine; T40: telmisartan 40 mg; T80: telmisartan 80 mg.

**Figure 2:**
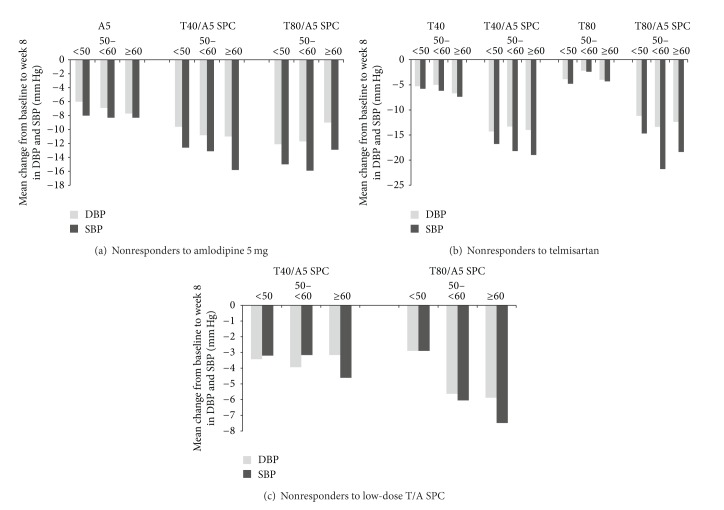
Change from baseline in DBP and SBP by age group in the three groups after 8 weeks' treatment. Baseline indicates reference baseline BP value, which was measured immediately before first dosing in the double-blind treatment period. A5: amlodipine 5 mg; BP: blood pressure; DBP: diastolic blood pressure; SBP: systolic blood pressure; SPC: single-pill combination; T/A: telmisartan/amlodipine; T40: telmisartan 40 mg; T80: telmisartan 80 mg.

In the individual studies, the adjusted mean treatment difference in DBP reduction was significantly higher with the T/A SPC than with either of the monotherapies (Table 3). The T80/A5 SPC also resulted in numerically greater reductions than the low-dose combination in nonresponders to T40/A5 (Table 3).  Figure 1 displays mean reductions in DBP and SBP from the reference baseline after 8 weeks of treatment for the three study groups. The advantages of the T/A SPC were consistent across age groups for the three study groups (Figures 2(a), 2(b), and 2(c)).

The BP goal attainment rates (<140/<90 mm Hg) at weeks 4 and 8 were higher with the T/A SPC in the nonresponders to amlodipine group and nonresponders to telmisartan group and also with the T80/A5 SPC compared with the T40/A5 SPC (Table 4). The percentage of patients achieving seated trough DBP and SBP goal and the response rates for DBP and SBP at week 8 were also higher with the T/A SPC than with the respective monotherapies (Table 4). The T80/A5 SPC resulted in higher DBP goal rates and DBP response rates at week 8 and in a higher SBP goal rate at week 4 compared with the T40/A5 combination (Table 4).

### 3.3. Safety

The overall incidence of AEs, discontinuation due to AEs, and incidence of peripheral edema in the individual studies are provided in Table 3. There were no deaths reported during the randomized treatment period in any of the five studies. Overall, 426 patients were exposed to A5 monotherapy, 245 patients to T40 or T80 monotherapy, and 897 patients to the T/A SPC (Table 5). The percentage of patients with any AEs was numerically lower with telmisartan and the T/A SPC than with amlodipine (Table 5). The occurrence of serious AEs was low and similar with amlodipine (angina pectoris *n* = 1 and cerebellar infarction *n* = 1) and the T/A SPC (Guillain-Barré syndrome *n* = 1, forearm fracture *n* = 1, cerebral artery occlusion *n* = 1, and clavicle fracture *n* = 1); no serious AEs were reported with telmisartan. The incidence of Guillain-Barré syndrome, cerebellar infarction, and clavicle fracture resulted in study discontinuation. Overall, less than 2% of patients in each group discontinued the study due to AEs.

The most common AEs that occurred in more than one patient on at least one of the treatments were nasopharyngitis, gastroenteritis, dizziness, peripheral edema, bronchitis, and back pain (Table 5). Nasopharyngitis was the AE with the highest incidence and occurred in a numerically higher percentage of patients on amlodipine (8.7%) and the T/A SPC (7.0%) than in patients on telmisartan (1.6%) (Table 5). Peripheral edema incidence was low and occurred in a similar percentage of patients on amlodipine (0.5%) and the T/A SPC (0.7%) and in no patients on telmisartan (Table 5).

## 4. Discussion

Patients with hypertension are a heterogeneous population encompassing many hypertensive phenotypes. Hence, combination therapy with pharmacologic action on two or more different physiologic sites is expected to be more effective, as it blocks the counterregulatory responses generally associated with monotherapy and its mostly single site of action [29]. Treatment with SPCs results in better BP control and long-term CV risk reduction [13, 30–32]. A prespecified substudy of the BP-lowering arm of the Anglo-Scandinavian Cardiac Outcomes Trial (ASCOT-BPLA) showed a RAS inhibitor plus a CCB to be a preferable and effective combination for South Asian patients [33].

In all the five studies reported here, the T/A SPC was more effective in lowering BP in Asian patients with hypertension whose BP was not adequately controlled with either monotherapy or low-dose combination therapy. The BP goal attainment rate and response rates at the end of 8 weeks were also higher with the T/A SPC than the respective monotherapies, and the DBP goal and response rate were higher with the T/A SPC than the low-dose T/A SPC. Similar beneficial results have been observed with the T/A SPC in other studies conducted in Asian patients. In the Cotalo study in Japanese patients whose BP was not adequately controlled with A5 monotherapy, 8 weeks' treatment with T40/A5 SPC significantly decreased the 24-hour mean and clinical BP, independent of administration time; results were similar between patients with or without metabolic syndrome [34]. In a prospective open-label study on Japanese patients with hypertension with BP uncontrolled on treatment with valsartan 80 mg/A5 or candesartan 8 mg/A5, switching to T40/A5 treatment resulted in a significant reduction in both mean clinic SBP and DBP at 4, 8, and 12 weeks, compared with valsartan 80 mg/A5 treatment suggesting more favorable CV outcomes with the T40/A5 combination [35]. Similar benefits were seen with T40/A5 in the subgroup of elderly patients; in addition, T40/A5 was found to significantly increase serum adiponectin levels, suggesting beneficial cardiometabolic effects in the elderly [36]. In a multicenter, open-label clinical trial in Chinese high-risk hypertensive patients with at least one CV risk factor, 96-week treatment with T/A was efficacious in reducing BP levels with acceptable goal rates and was well tolerated [37].

The results reported here are consistent with the results from international studies conducted in patients with mild-to-moderate or severe hypertension not controlled on amlodipine monotherapy [38–41] and suggest similar benefits with the T/A combination in Asian patients. In international studies, the T/A combination compared with the respective monotherapies has also been shown to provide superior 24-hour BP lowering in patients with mild-to-moderate hypertension [42] and significantly greater BP reductions and higher BP goal and response rates in patients with severe hypertension [43]. Planned and *post hoc* pooled analysis of data from clinical trials have shown the T/A SPC to be efficacious and well tolerated in hypertensive patients with added risk factors, including obesity, diabetes, metabolic syndrome, renal impairment, and elevated SBP [44–46].

Overall, double-blind treatment for 8 weeks with either monotherapy or with a T/A SPC was well tolerated in the five included studies. The safety and tolerability data obtained after 8 weeks of double-blind, randomized treatment were consistent with the known safety and tolerability of telmisartan and amlodipine or the T/A SPC, and no clinically important differences were noted. In previous studies, T/A SPC treatment was associated with a lower incidence of peripheral edema than with amlodipine monotherapy [38–41]. In this analysis of studies on Asian patients, the incidence of peripheral edema was low and was reported in less than 1% of patients on amlodipine monotherapy and the T/A SPC and in no patients receiving telmisartan monotherapy. The overall lower incidence of edema observed in this study could be related to the differences between ethnic groups in risks for certain AEs.

The studies reported here included only Japanese, Chinese, Malaysian, and Philippine patients, which may limit the applicability of the findings to patients from other Asian countries. Also, the controlled nature of the studies limits generalization of the results to those categories of patients who were excluded from these studies.

## 5. Conclusions

In Asian patients whose BP is not adequately controlled with telmisartan or amlodipine monotherapy, 8 weeks' treatment with a T/A combination results in greater BP reduction and higher DBP and SBP goal and response rates. In Asian patients whose BP is not adequately controlled with the T40/A5 SPC, 8 weeks' treatment with the T80/A5 SPC results in greater BP reduction and DBP goal and response rates. The safety and tolerability profile of the T/A SPC is comparable to that of the respective monotherapies and consistent with that reported in previous studies.

## Figures and Tables

**Table 1:** Details of included trials.

	A5 nonresponder study 1	A5 nonresponder study 2	T40 nonresponder study	T80 nonresponder study	Nonresponder to low-dose T/A combination study
Trial identifier	NCT00558064	NCT01103960	NCT00550953	NCT01222520	NCT01286558

Reference	Data on file	Zhu et al [28]	Data on file	Data on file	Data on file

Trial duration	October 10, 2007–September 27, 2008	July 28, 2010–August 27, 2011	October 15, 2007–July 19, 2008	October 23, 2010–June 18, 2011	January 22, 2011–October 15, 2011

Study sites	41 sites in Japan	16 investigative sites—12 sites in the People's Republic of China, 2 sites in Malaysia, and 2 sites in Philippines	5 sites in Japan	4 sites in Japan	8 sites in Japan

BP inclusion criteria, start of open-label run-in period	DBP ≥95 and ≤114 mm Hg SBP ≥140 and ≤200 mm Hg	DBP ≥95 if on antihypertensive treatment, or ≥100 mm Hg if treatment is naïve	DBP ≥95 and ≤114 mm Hg SBP ≥140 and ≤200 mm Hg	DBP ≥90 and ≤114 mm Hg in patients on antihypertensive drugs; DBP ≥95 and ≤114 mm Hg in treatment-naïve patients SBP ≥140 and ≤200 mm Hg	DBP ≥90 and ≤114 mm Hg in patients on antihypertensive drugs; DBP ≥95 and ≤114 mm Hg in treatment-naïve patients SBP ≤200 mm Hg

BP inclusion criteria, start of 8-week double-blind treatment period	DBP ≥90 and ≤114 mm Hg SBP ≤200 mm Hg	DBP ≥90 mm Hg	DBP ≥90 and ≤114 mm Hg SBP ≤200 mm Hg	DBP ≥90 and ≤114 mm Hg SBP ≤200 mm Hg	DBP ≥80 and ≤114 mm Hg SBP ≤200 mm Hg

BP exclusion criteria	SBP/DBP >200/>114 mm Hg at screening, at the start of the open-label run-in period or double-blind treatment period SBP/DBP >200/>114 mm Hg or DBP <90 mm Hg, 2 weeks after the start of the open-label run-in period	BP ≥ 200/120 mm Hg at screening or during the run-in period; ≥180/120 mm Hg at the end of the run-in period	SBP/DBP >200/>114 mm Hg at screening, at the start of the open-label run-in period or double-blind treatment period DBP <90 mm Hg at the start of the open-label run-in period		

Duration and treatment during open-label run-in period	A5 once daily for 6 weeks	A5 once daily for 6 weeks	T20 once daily for 2 weeks then T40 once daily for 4 weeks	T20 for 2 weeks; T40 for 2 weeks; T80 for 4 weeks	T20 for 2 weeks; T40 for 4 weeks; T40/A5 6 weeks

*N*, run-in period	636	381	357	197	292

Duration of double-blind period	8 weeks	8 weeks	8 weeks	8 weeks	8 weeks

Double-blind treatment groups	A5; T20 + A5 for 2 weeks; T40/A5 SPC for 6 weeks	A5; T80/A5 SPC	T40; T40/A5 SPC	T80; T80/A5 SPC	T40/A5 SPC; T80/A5 SPC

*N*, randomized groups	262; 269	164; 160	158; 156	87; 87	113; 112

A5: amlodipine 5 mg; BP: blood pressure; DBP: diastolic blood pressure; NCT: National Clinical Trial; SBP: systolic blood pressure; SPC: single-pill combination; T20: telmisartan 20 mg; T40: telmisartan 40 mg; T80: telmisartan 80 mg; T/A: telmisartan plus amlodipine.

DBP and SBP are mean seated values.

**Table 2:** Demographics and baseline characteristics (full analysis set).

	Nonresponders to A5			Nonresponders to T40 or T80				Nonresponders to low-dose T/A		Total
	A5 (*n* = 416)	T40/A5 SPC (*n* = 263)	T80/A5 SPC (*n* = 155)	T40 (*n* = 158)	T40/A5 SPC (*n* = 153)	T80 (*n* = 86)	T80/A5 SPC (*n* = 87)	T40/A5 SPC (*n* = 112)	T80/A5 SPC (*n* = 112)	*N* = 1542
Age, mean (SD)	54.6 (10.0)	57.0 (9.6)	52.4 (8.7)	55.5 (10.2)	55.6 (9.1)	54.8 (8.7)	54.5 (9.0)	52.8 (9.4)	54.6 (8.4)	54.9 (9.5)
Age group, *n* (%)										
<50 years	124 (29.8)	63 (24.0)	60 (38.7)	43 (27.2)	35 (22.9)	24 (27.9)	28 (32.2)	45 (40.2)	35 (31.3)	457 (29.6)
50–<60 years	159 (38.2)	100 (38.0)	65 (41.9)	62 (39.2)	66 (43.1)	34 (39.5)	31 (35.6)	40 (35.7)	49 (43.8)	606 (39.3)
≥60 years	133 (32.0)	100 (38.0)	30 (19.4)	53 (33.5)	52 (34.0)	28 (32.6)	28 (32.2)	27 (24.1)	28 (25.0)	479 (31.1)
BMI, mean (SD)	25.7 (3.9)	25.5 (3.2)	26.1 (3.1)	25.7 (4.0)	25.3 (3.6)	26.4 (4.1)	25.6 (4.0)	26.2 (4.0)	26.1 (4.2)	25.8 (3.7)
Sex, *n* (%)										
Female	147 (35.3)	71 (27.0)	78 (50.3)	35 (22.2)	42 (27.5)	12 (14.0)	20 (23.0)	24 (21.4)	23 (20.5)	452 (29.3)
Male	269 (64.7)	192 (73.0)	77 (49.7)	123 (77.8)	111 (72.5)	74 (86.0)	67 (77.0)	88 (78.6)	89 (79.5)	1090 (70.7)
Race, *n* (%)										
Asian	416 (100.0)	263 (100.0)	155 (100.0)	158 (100)	153 (100)	86 (100)	87 (100)	112 (100)	112 (100)	1542 (100)
Diabetes										
No	357 (85.8)	213 (81.0)	137 (88.4)	147 (93.0)	141 (92.2)	77 (89.5)	80 (92.0)	105 (93.8)	105 (93.8)	1362 (88.3)
Yes	59 (14.2)	50 (19.0)	18 (11.6)	11 (7.0)	12 (7.8)	9 (10.5)	7 (8.0)	7 (6.3)	7 (6.3)	180 (11.7)
Mean (SD) baseline SBP, mm Hg	145.7 (10.7)	144.6 (11.8)	146.4 (12.2)	144.8 (13.4)	145.7 (12.2)	144.3 (14.5)	143.7 (13.8)	134.5 (13.6)	133.7 (10.6)	143.6 (12.7)
Mean (SD) baseline DBP, mm Hg	96.5 (5.6)	95.9 (5.0)	97.2 (5.5)	96.6 (6.0)	96.8 (5.3)	98.5 (6.7)	98.0 (6.0)	90.7 (8.0)	90.1 (6.7)	95.8 (6.3)
Baseline PR, mean (SD)	71.4 (9.4)	70.6 (8.8)	70.7 (7.6)	69.1 (8.5)	68.8 (7.7)	71.2 (9.8)	68.1 (8.3)	71.1 (9.8)	71.7 (9.3)	70.5 (8.9)
Disease duration, mean (SD)	7.5 (8.0)	6.8 (7.8)	7.7 (8.9)	7.1 (7.5)	7.8 (7.7)	8.6 (6.1)	8.3 (6.4)	7.8 (7.6)	6.6 (6.8)	7.5 (8.0)

A5: amlodipine 5 mg; BMI: body mass index; DBP: diastolic blood pressure; PR: pulse rate; SBP: systolic blood pressure; SD: standard deviation; SPC: single-pill combination; T40: telmisartan 40 mg; T80: telmisartan 80 mg; T/A: telmisartan plus amlodipine.

The number of patients included in this analysis may differ from those of the individual study publications, due to differences in statistical approach for the individual studies.

**Table 3:** Efficacy and safety results of individual studies.

	A5 nonresponder study 1		A5 nonresponder study 2		T40 nonresponder study		T80 nonresponder study		Nonresponder to low-dose combination study	
Trial identifier	NCT00558064		NCT01103960		NCT00550953		NCT01222520		NCT01286558	
Reference	Data on file		Zhu et al., [28]		Data on file		Data on file		Data on file	
Double-blind treatment groups	A5	T20 + A5 for 2 weeks; T40/A5 SPC for 6 weeks	A5	T80/A5 SPC	T40	T40/A5 SPC	T80	T80/A5 SPC	T40/A5 SPC	T80/A5 SPC
*N*, randomized	262	269	164	160	158	156	87	87	113	112
Adjusted mean treatment difference in DBP*, 95% CI		5.1 (4.0–6.2)**		2.4 (0.7–4.1)^†^		8.0 (6.4–9.6)**		9.1 (7.1–11.2)**		1.5 (−0.2, 3.4)
Overall incidence of adverse events, *n* (%)	78 (29.8)	81 (30.1)	33 (20.1)	33 (20.6)	35 (22.2)	47 (30.1)	14 (16.1)	12 (13.8)	26 (23.0)	19 (17.0)
Incidence of peripheral edema, *n* (%)	1 (0.4%)	0 (0.0)	1 (0.6%)	1 (0.6%)	0 (0.0)	0 (0.0)	0 (0.0)	0 (0.0)	2 (1.8)	2 (1.8)
Discontinuation due to adverse events, *n* (%)	2 (0.8%)	3 (1.1%)	2 (1.2)	1 (0.6)	3 (1.9)	3 (1.9)	1 (1.1)	0 (0.0)	1 (0.9)	1 (0.9)

A5: amlodipine 5 mg; CI: confidence interval; DBP: diastolic blood pressure; NA: not applicable; NCT: National Clinical Trial; SE: standard error; SPC: single-pill combination; T20: telmisartan 20 mg; T40: telmisartan 40 mg; T80: telmisartan 80 mg.

*The treatment effect (i.e., the difference between treatment groups in reduction from the reference baseline in mean seated DBP at trough after 8 weeks of double-blind treatment) was estimated by the least squares mean and its 95% CI.

**P < 0.0001, ^†^ *P* < 0.01.

**Table 4:** Blood pressure goal rate and response rate at weeks 4 and 8 in the three groups (full analysis set).

	Nonresponders to A5			Nonresponders to T40 or T80				Nonresponders to low-dose T/A	
	A5 (*n* = 416)	T40/A5 SPC (*n* = 263)	T80/A5 SPC (*n* = 155)	T40 (*n* = 158)	T40/A5 SPC (*n* = 153)	T80 (*n* = 86)	T80/A5 SPC (*n* = 87)	T40/A5 SPC (*n* = 112)	T80/A5 SPC (*n* = 112)
BP < 140/90 mm Hg									
Week 4, *n* (%)	158 (38.0)	155 (58.9)	77 (49.7)	55 (34.8)	97 (63.4)	17 (19.8)	46 (52.9)	61 (54.5)	64 (57.1)
Week 8, *n* (%)	160 (38.5)	163 (62.0)*	100 (64.5)	59 (37.3)	113 (73.9)	20 (23.3)	59 (67.8)	68 (60.7)	71 (63.4)

SBP < 140 mm Hg									
Week 4, *n* (%)	232 (55.8)	194 (73.8)	103 (66.5)	88 (55.7)	121 (79.1)	37 (43.0)	68 (78.2)	83 (74.1)	86 (76.8)
Week 8, *n* (%)	246 (59.1)	205 (77.9)*	119 (76.8)	87 (55.1)	113 (86.9)	43 (50.0)	73 (83.9)	90 (80.4)	89 (79.5)

DBP < 90 mm Hg									
Week 4, *n* (%)	211 (50.7)	179 (68.1)	95 (61.3)	67 (42.4)	106 (69.3)	19 (22.1)	49 (56.3)	73 (65.2)	69 (61.6)
Week 8, *n*(%)	206 (49.5)	179 (68.1)*	112 (72.3)	74 (46.8)	120 (78.4)	23 (26.7)	61 (70.1)	74 (66.1)	78 (69.6)

SBP < 140 or reduction of ≥20 mm Hg**									
Week 4, *n* (%)	253 (60.8)	202 (76.8)	124 (80.0)	93 (58.9)	123 (80.4)	37 (43.0)	71 (81.6)	83 (74.8)	86 (76.8)
Week 8, *n* (%)	270 (64.9)	209 (79.5)*	131 (84.5)	92 (58.2)	137 (89.5)	44 (51.2)	77 (88.5)	92 (82.1)	90 (80.4)

DBP < 90 or reduction of ≥10 mm Hg									
Week 4, *n* (%)	236 (56.7)	190 (72.2)	108 (69.7)	73 (46.2)	118 (77.1)	21 (24.4)	56 (64.4)	77 (69.4)	70 (62.5)
Week 8, *n* (%)	230 (55.3)	196 (74.5)*	124 (80.0)	79 (50.0)	130 (85.0)	26 (30.2)	70 (80.5)	76 (67.9)	79 (70.5)

The number of patients included in this analysis may differ from those of the individual study publications, due to differences in statistical approach for the individual studies.

*The data are for the 6 weeks' duration when patients were on T40/A5 therapy and do not include the 2 weeks when patients were on T20/A5 therapy; **in A5 nonresponder study 2, SBP response was defined as (<140 mmHg and/or reduction from baseline ≥15 mmHg).

A5: amlodipine 5 mg; BP: blood pressure; DBP: diastolic blood pressure; SBP: systolic blood pressure; SPC: single-pill combination; T20: telmisartan 20 mg; T40: telmisartan 40 mg; T80: telmisartan 80 mg; T/A: telmisartan plus amlodipine.

**Table 5:** Safety results of 8 weeks' treatment with T/A combination or the respective monotherapies.

	Amlodipine monotherapy	Telmisartan monotherapy	T/A SPC therapy
Patients treated, *n*	426	245	897
Total exposure (patient-years)	66.6	31.6	151.8
Patients with any AE, *n* (%)	111 (26.1)	49 (20.0)	218 (24.3)
Patients with drug-related AEs, *n* (%)	9 (2.1)	3 (1.2)	24 (2.7)
Patients with SAE, *n* (%)	2 (0.5)	0 (0)	4 (0.4)
Patients with AEs leading to discontinuation, *n* (%)	4 (0.9)	4 (1.6)	9 (1.0)

Most common AEs that occurred in more than one patient on at least one of the treatments, *n* (%)			
Nasopharyngitis	37 (8.7)	4 (1.6)	63 (7.0)
Gastroenteritis	4 (0.9)	5 (2.0)	4 (0.4)
Dizziness	4 (0.9)	0 (0)	8 (0.9)
Peripheral edema	2 (0.5)	0 (0)	6 (0.7)
Bronchitis	2 (0.5)	0 (0)	5 (0.6)
Back pain	2 (0.5)	1 (0.4)	3 (0.3)

AE: adverse event; SAE: serious adverse event; SPC: single-pill combination; T/A: telmisartan plus amlodipine.

The number of patients included in this analysis may differ from those of the individual study publications, due to differences in statistical approach for the individual studies.

## References

[1] Martiniuk AL, Lee CM, Lawes CMM (2007). Hypertension: its prevalence and population-attributable fraction for mortality from cardiovascular disease in the Asia-Pacific region. *Journal of Hypertension*.

[2] Ueshima H, Iimura O, Iida M, Okayama A, Sawai K, Minowa M (2003). Impact of elevated blood pressure on mortality from all causes, cardiovascular diseases, heart disease and stroke among Japanese: 14 year follow-up of randomly selected population from Japanese—nippon data 80. *Journal of Human Hypertension*.

[3] Miura K (2011). Epidemiology and prevention of hypertension in Japanese: how could Japan get longevity?. *EPMA Journal*.

[4] Okayama A, Kadowaki T, Okamura T, Hayakawa T, Ueshima H (2006). Age-specific effects of systolic and diastolic blood pressures on mortality due to cardiovascular diseases among Japanese men (NIPPON DATA80). *Journal of Hypertension*.

[5] Gu D, Kelly TN, Wu X (2008). Blood pressure and risk of cardiovascular disease in chinese men and women. *The American Journal of Hypertension*.

[6] Murakami Y, Hozawa A, Okamura T, Ueshima H (2008). Relation of blood pressure and all-cause mortality in 180, 000 japanese participants pooled analysis of 13 cohort studies. *Hypertension*.

[7] Ma YQ, Mei WH, Yin P, Yang XH, Rastegar SK, Yan JD (2013). Prevalence of hypertension in Chinese cities: a meta-analysis of published studies. *PLoS ONE*.

[8] Sheng CS, Liu M, Kang YY (2013). Prevalence, awareness, treatment and control of hypertension in elderly Chinese. *Hypertension Research*.

[9] Kjeldsen SE, Messerli FH, Chiang CE, Meredith PA, Liu L (2012). Are fixed-dose combination antihypertensives suitable as first-line therapy?. *Current Medical Research and Opinion*.

[10] Gradman AH, Basile JN, Carter BL, Bakris GL (2010). Combination therapy in hypertension. *Journal of the American Society of Hypertension*.

[11] Chobanian AV, Bakris GL, Black HR (2003). Seventh report of the joint national committee on prevention, detection, evaluation, and treatment of high blood pressure. *Hypertension*.

[12] Mancia G, Fagard R, Narkiewicz K (2013). ESH/ESC Guidelines for the management of arterial hypertension: the Task Force for the management of arterial hypertension of the European Society of Hypertension (ESH) and of the European Society of Cardiology (ESC). *European Heart Journal*.

[13] Bangalore S, Ley L (2012). Improving treatment adherence to antihypertensive therapy: the role of single-pill combinations. *Expert Opinion on Pharmacotherapy*.

[14] Sherrill B, Halpern M, Khan S, Zhang J, Panjabi S (2011). Single-pill vs free-equivalent combination therapies for hypertension: a meta-analysis of health care costs and adherence. *Journal of Clinical Hypertension*.

[15] Bangalore S, Kamalakkannan G, Parkar S, Messerli FH (2007). Fixed-dose combinations improve medication compliance: a meta-analysis. *The American Journal of Medicine*.

[16] Akazawa M, Fukuoka K (2013). Economic impact of switching to fixed-dose combination therapy for Japanese hypertensive patients: a retrospective cost analysis. *BMC Health Services Research*.

[17] Hess G, Hill J, Lau H, Dastani H, Chaudhari P (2008). Medication utilization patterns and hypertension-related expenditures among patients who were switched from fixed-dose to free-combination antihypertensive therapy. *P and T*.

[18] Gupta AK, Arshad S, Poulter NR (2010). Compliance, safety, and effectiveness of fixed-dose combinations of antihypertensive agents: a meta-analysis. *Hypertension*.

[19] Mancia G, Laurent S, Agabiti-Rosei E (2009). Reappraisal of European guidelines on hypertension management: a European Society of Hypertension Task Force document. *Journal of Hypertension*.

[20] Ogihara T, Kikuchi K, Matsuoka H (2009). The Japanese Society of Hypertension Guidelines for the Management of Hypertension (JSH 2009). *Hypertension Research*.

[21] Neutel JM (2013). Treatment algorithms for hypertension: a practical approach. *Clinical Practice*.

[22] Rubio-Guerra AF, Castro-Serna D, Barrera CI, Ramos-Brizuela LM (2009). Current concepts in combination therapy for the treatment of hypertension: combined calcium channel blockers and RAAS inhibitors. *Integrated Blood Pressure Control*.

[23] Mallat SG (2012). Which is the preferred angiotensin II receptor blocker-based combination therapy for blood pressure control in hypertensive patients with diabetic and non-diabetic renal impairment?. *Cardiovascular Diabetology*.

[24] Yusuf S, Teo KK, Pogue J (2008). Telmisartan, ramipril, or both in patients at high risk for vascular events. *The New England Journal of Medicine*.

[25] Burnier M (2009). Telmisartan: a different angiotensin II receptor blocker protecting a different population?. *Journal of International Medical Research*.

[26] Galzerano D, Capogrosso C, di Michele S (2010). New standards in hypertension and cardiovascular risk management: focus on telmisartan. *Vascular Health and Risk Management*.

[27] Dans AL, Teo K, Gao P (2010). In a subgroup of high-risk asians, telmisartan was non-inferior to ramipril and better tolerated in the prevention of cardiovascular events. *PLoS ONE*.

[28] Zhu D, Gao P, Holtbruegge W, Huang C (2014). A randomized, double-blind study to evaluate the efficacy and safety of a singlepill combination of telmisartan 80 mg/amlodipine 5 mg versus amlodipine 5 mg in hypertensive Asian patients. *Journal of International Medical Research*.

[29] Sever PS, Messerli FH (2011). Hypertension management 2011: optimal combination therapy. *European Heart Journal*.

[30] Egan BM, Bandyopadhyay D, Shaftman SR, Wagner CS, Zhao Y, Yu-Isenberg KS (2012). Initial monotherapy and combination therapy and hypertension control the first year. *Hypertension*.

[31] Gradman AH, Parise H, Lefebvre P, Falvey H, Lafeuille MH, Duh MS (2013). Initial combination therapy reduces the risk of cardiovascular events in hypertensive patients: a matched cohort study. *Hypertension*.

[32] Corrao G, Nicotra F, Parodi A (2011). Cardiovascular protection by initial and subsequent combination of antihypertensive drugs in daily life practice. *Hypertension*.

[33] Gupta AK, Poulter NR, Dobson J (2010). Ethnic differences in blood pressure response to first and second-line antihypertensive therapies in patients randomized in the ASCOT trial. *The American Journal of Hypertension*.

[34] Ohishi M, Kawai T, Hayashi N (2013). Effect of tablets with a combination of telmisartan and amlodipine on patients with hypertension: the Cotalo study. *Hypertension Research*.

[35] Bekki H, Yamamoto K, Sone M (2010). Efficacy of combination therapy with telmisartan plus amlodipine in patients with poorly controlled hypertension. *Oxidative Medicine and Cellular Longevity*.

[36] Bekki H, Yamamoto K, Sone M (2011). Beneficial cardiometabolic actions of telmisartan plus amlodipine therapy in elderly patients with poorly controlled hypertension. *Clinical Cardiology*.

[37] Ma L, Wang W, Zhao Y (2012). Combination of amlodipine plus angiotensin receptor blocker or diuretics in high-risk hypertensive patients: a 96-week efficacy and safety study. *The American Journal of Cardiovascular Drugs*.

[38] Littlejohn TW, Majul CR, Olvera R (2009). Results of treatment with telmisartan-amlodipine in hypertensive patients. *The Journal of Clinical Hypertension*.

[39] Littlejohn TW, Majul CR, Olvera R (2009). Telmisartan plus amlodipine in patients withmoderate or severe hypertension: results from a subgroup analysis of a randomized, placebo-controlled, 
parallel-group, 4 × 4 factorial study. *Postgraduate Medicine*.

[40] Neldam S, Lang M, Jones R (2011). Telmisartan and amlodipine single-pill combinations vs amlodipine monotherapy for superior blood pressure lowering and improved tolerability in patients with uncontrolled hypertension: results of the TEAMSTA-5 study. *Journal of Clinical Hypertension*.

[41] Neldam S, Edwards C, Jones R (2011). Switching patients with uncontrolled hypertension on amlodipine 10mg to single-pill combinations of telmisartan and amlodipine: results of the TEAMSTA-10 study. *Current Medical Research and Opinion*.

[42] White WB, Littlejohn TW, Majul CR (2010). Effects of telmisartan and amlodipine in combination on ambulatory blood pressure in stages 1-2 hypertension. *Blood Pressure Monitoring*.

[43] Neutel JM, Mancia G, Black HR (2012). Single-pill combination of telmisartan/amlodipine in patients with severe hypertension: results from the TEAMSTA severe HTN study. *The Journal of Clinical Hypertension*.

[44] Guthrie RM, Dahlöf B, Jamerson KA (2011). Efficacy and tolerability of telmisartan plus amlodipine in added-risk hypertensive patients. *Current Medical Research and Opinion*.

[45] Ley L, Schumacher H (2013). Telmisartan plus amlodipine single-pill combination for the management of hypertensive patients with a metabolic risk profile (added-risk patients). *Current Medical Research and Opinion*.

[46] Sharma AM, Bakris G, Neutel JM (2012). Single-pill combination of telmisartan/Amlodipine versus amlodipine monotherapy in diabetic hypertensive patients: an 8-week randomized, parallel-group, double-blind trial. *Clinical Therapeutics*.

